# Association of high body mass index with development of interstitial fibrosis in patients with IgA nephropathy

**DOI:** 10.1186/s12882-018-1164-2

**Published:** 2018-12-29

**Authors:** Changwei Wu, Amanda Y. Wang, Guisen Li, Li Wang

**Affiliations:** 1 Renal Department and Nephrology Institute, Sichuan Provincial People’s Hospital, School of Medicine, University of Electronic Science and Technology of China, No. 32 West 2nd Duan, 1st Circle Road, Qingyang District, Chengdu, Sichuan 610072 People’s Republic of China; 2 Renal and metabolic division, The George institute for global health, Sydney, Australia; 3 The Faculty of medicine and health sciences, Macquarie University, Sydney, Australia

**Keywords:** IgA nephropathy, Overweight, Body mass index, Interstitial fibrosis, Progression, Renal outcomes

## Abstract

**Background:**

The worldwide prevalence of obesity is increasing. Obesity is associated with a variety of chronic diseases, including chronic kidney disease. Several studies suggested that body mass index (BMI) could be an independent risk factor for progression of IgA nephropathy (IgAN). However, whether high BMI is associated with progression of IgAN remains uncertain.

**Methods:**

This retrospective study included patients with biopsy proven IgAN from 2006 to 2017 in Sichuan Provincial People’s Hospital. BMI was categorized according to the WHO Asian guideline: underweight (< 18.5 kg/m^2^), normal weight (18.5-25 kg/m^2^), overweight (25-28 kg/m^2^) and obese (≥28 kg/m^2^). The main outcome was development of end-stage renal disease (ESRD) or a decline in eGFR by at least 30%. The association of BMI and IgAN progression was determined by propensity-score-matched cohort analysis.

**Results:**

Four hundred eighty one patients with IgAN were finally enrolled in this study. The mean age was 37 ± 11 years and 40.3% were men. There was no significant difference in clinical and pathological characteristics among the four-group patients categorized by BMI. After matching with propensity scores, no significant correlation between BMI and renal outcomes was seen. However, compared with the reference group (18.5≦BMI≦25 kg/m^2^), being overweight (odd ratio [OR], 2.28; 95%CI: 1.06–4.88; *P* = 0.034) and obese (OR, 3.43; 95%CI: 1.06–11.04; *P* = 0.039) was associated with a high risk of interstitial fibrosis. In the cross figure demonstrating the association of BMI subgroup and interstitial fibrosis on renal outcomes, ORs of interstitial fibrosis groups were higher than those of no interstitial fibrosis. Compared with other BMI subgroups, patients with 18.5-25 kg/m^2^ had lowest ORs.

**Conclusions:**

High BMI and interstitial fibrosis were associated with progression of IgAN. Interstitial fibrosis appears to be common in IgAN patients with elevated BMI.

## Backgroud

Obesity is a growing global healthcare issue [1, 2]. In 2015, approximately 12% adults were obese worldwide with the highest incidence in early adulthood [3]. China, notably, had the highest numbers of obese children and adults, compared with other countries [3]. Recent epidemiologic studies suggested that high body-mass index (BMI) was one of the risk factors of chronic diseases, including chronic kidney disease (CKD), cardiovascular disease, cancer [4] and musculoskeletal disorders [5]. Chronic kidney disease was shown to be the second common cause of obesity-related mortality and disability-adjusted life-years, just after cardiovascular disease [3].

Over the years, several studies have been conducted to investigate the effects of BMI on kidney diseases. Sankar et al. [6] found that a higher BMI were associated with lower risk for cardiovascular and non-cardiovascular related death in CKD. Stenvinkel et al. [7] suggested that obesity could increase the risk for CKD and accelerate the progression of CKD to end stage renal disease (ESRD). In addition, obesity could also lead to CKD, characterized by proteinuria and adaptive focal segmental glomerulosclerosis in renal biopsy [8]. However, there was paucity of studies investigating relationship between BMI and IgA nephropathy (IgAN). To date, all three studies in this field were performed in Japan [9–11], showing high BMI can serve as a risk factor for progress in IgAN. Berthoux et al. [12, 13] thought that IgAN patients with high BMI had worse clinical outcome, although BMI did not show a direct effect on worse clinical outcomes. On the contrary, Nan et al. [14] found that underweight, not overweight, was an independent risk factor for loss of renal function in IgAN.

To address the inconsistent findings based on current literatures, we conducted a retrospective cohort study using the propensity-score-matched analysis to systematically evaluate the effects of BMI on IgAN.

## Methods

### Study design

This was a retrospective study used the propensity-score-matched cohort analysis. The cohort included participants from a general hospital covering more than 80 million people in Southwest China. The protocol was approved by the Ethics Committee of Sichuan Provincial People’s Hospital, and informed consent was obtained prior to the study.

The inclusion criteria were adult patients with renal biopsy-proven primary IgAN with available data ontheir BMI, laboratory data such as serum creatinine, and proteinuria, detailed information on renal biopsy and availability of at the biopsy slides for further review if required. All information was obtained from the renal treatment system (RTS) database. Exclusion criteria included patients with secondary causes of glomerulonephritis such as systemic diseases including diabetes, chronic liver disease and systemic lupus erythematosus (SLE), secondary glomerular diseases, Henoch-Schonlein purpura, kidney transplantation, and pregnancy. Patients whose follow-up duration was less than 3 months were also excluded in the follow up analysis.

### Definitions

Pathology slides were reviewed by an expert renal pathologist and scored according to the baseline Oxford classification [15]. BMI was calculated as (weight in kilograms)/ (height in meters) ^2^ and categorized according to the WHO Asian guideline [16]: underweight (< 18.5 kg/m^2^), normal weight (18.5-25 kg/m^2^, reference category), overweight (25-28 kg/m^2^) and obesity (≥28 kg/m^2^). We estimated the estimated glomerular filtration rate (eGFR) by using the CKD-EPI equation [17]. The score of interstitial fibrosis was made according to the estimated percentage: none (0%), mild (< 30%), moderate (30–60%) and severe (> 60%).

The renal outcomes and all cause mortality were evaluated. Renal outcomes included 1)patients whose eGFR was 30% decreased [18] from baseline values; 2) patients who started hemodialysis or peritoneal dialysis; 3) patients who accepted kidney transplantation. The duration of follow-up was calculated from the time of renal biopsy to the last follow-up.

### Statistical analyses

Categorical variables, such as the presence of interstitial fibrosis, were presented as frequencies and percentages which were compared using the chi-squared test. Laboratory characteristics were presented as means ± SD or median and compared using the Kruskal–Wallis H test for normally and non-normally distributed variables.

Propensity-score matching was performed using the custom dialog in SPSS, where 1:1 nearest neighbor matching without replacement, and a caliper definition of 0.1, was used to determine the groups for the analysis of the effect of BMI on renal progression and interstitial fibrosis. We performed 3 matching procedures between the reference category (18.5-25 kg/m^2^) and other BMI categories to identify patients with a similar propensity for the reference BMI, in terms of their age, sex, eGFR, and proteinuria. In addition, we conducted two matched cohorts, follow-up matched cohort based on 295 follow-up participants and baseline matched cohort based on initially 481 participants.

The effect of BMI on progression of IgAN was tested using Kaplan-Meier survival analysis and logistic regression based on the follow-up matched cohort. The effect of BMI on progression of interstitial fibrosis was assessed using binary logistic regression, based on the baseline matched cohort. Finally, binary logistic regression was used to explore the effect of interstitial fibrosis on progression of IgAN in four cohorts of different BMI category, separately.

All analyses were carried out using SPSS 22 software version (SPSS, Inc., Chicago, IL). *P* value being two tailed and odds ratio (OR) with a 95% confidence interval (CI) were both indicated the strength. *P* value< 0.05 was considered statistically significant.

## Results

### Participants

**Fig. 1:**
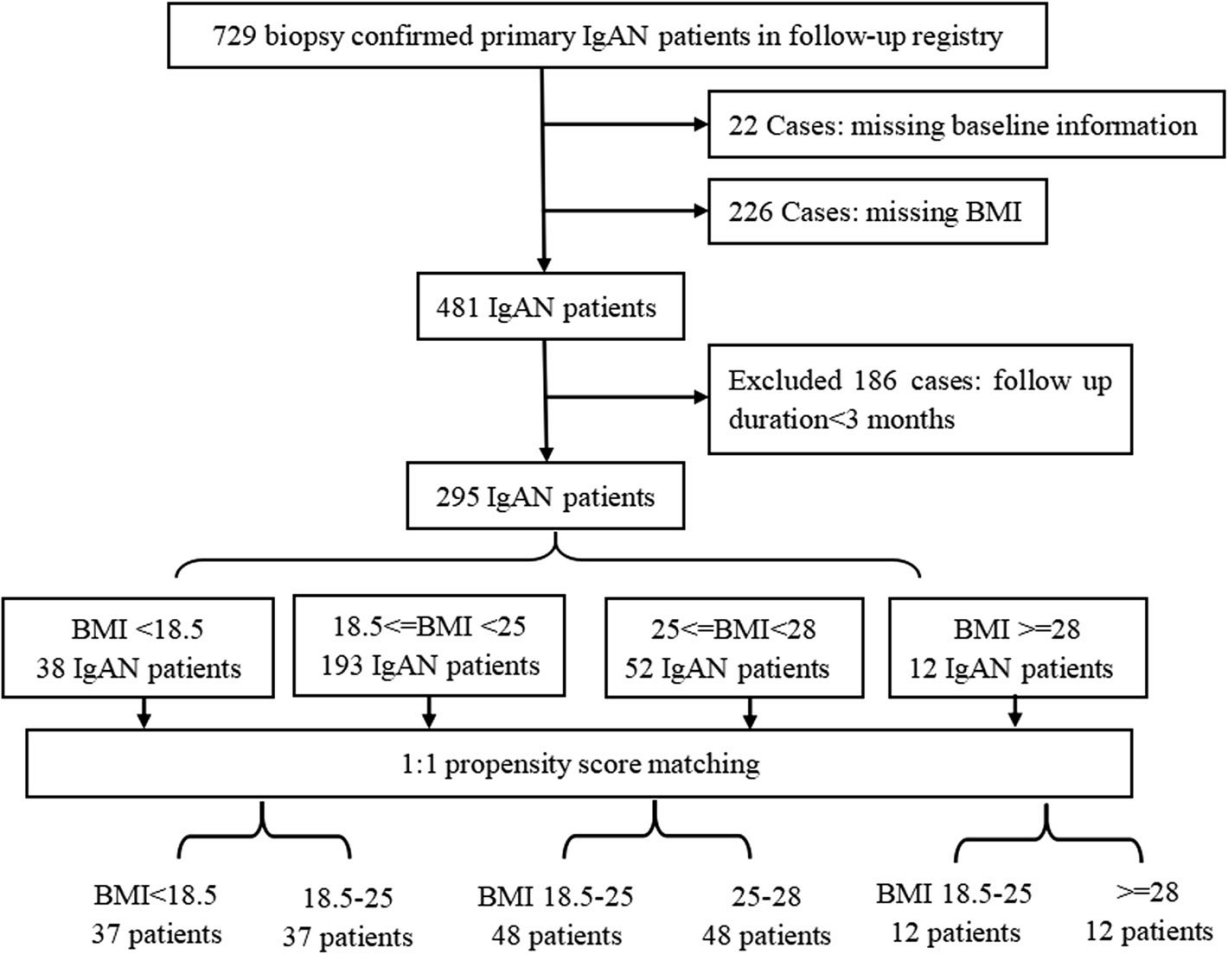
Study selection flow chart. In this figure, the matched cohort was follow-up matched cohort

Our cohort initially included 729 IgAN individuals who had biopsy proven primary IgAN. Then 248 subjects were excluded due to lack of baseline information. In total, 481 IgAN individuals were recruited in the study. In the follow-up analysis, 188 patients were excluded due to either lack of follow-up information or shorter followed-up than 3 months. Finally, 295 IgAN patients were included in the follow-up study and had the propensity-score matching performed. (Fig. 1).

### Cohort description and baseline characteristics

**Fig. 2:**
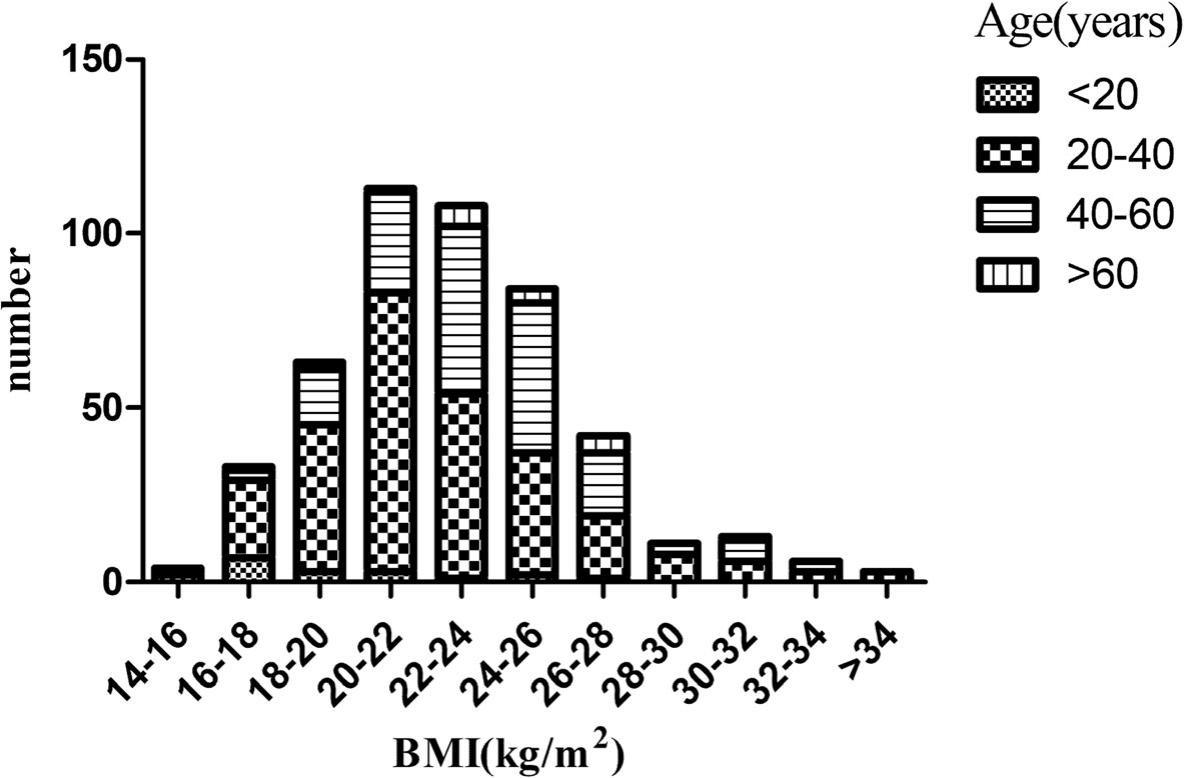
Distribution of BMI in 481 patients with IgAN

For all patients, the peak BMI was 20-22 kg/m^2^, which was shifted to the left (Fig. 2). The lowest and highest BMI were 14.62 and 39.18 kg/m^2^, respectively. The mean age of the whole cohort was 37 ± 11 years and females were predominant, accounting for 59.87% of the cohort. The baseline eGFR was 85.92 ± 32.28 ml/min per 1.73 m^2^ and 24 h urinary protein excretion was 2.06 ± 2.26 g/24 h (Table 1). Upon the time of diagnosis, 50 patients were underweight, 314 patients were having normal weight, 83 patients were overweight and 33 patients were obese. Male patients (*P* = 0.004) and elderly people appeared to have higher BMI (*P* = 3.6E-11). In addition, increased serum uric acid (UA) (*P* = 0.03) and haemoglobin levels (*P* = 0.002) and less gross hematuria (*P* = 1.1E-5) were associated with higher BMI (Table 1).

**Table 1:** Demographic, clinical, and laboratory characteristics at renal biopsy

	Total	BMI < 18.5 kg/m^2^	18.5 ≤ BMI<25 kg/m^2^	25 ≤ BMI<28 kg/m^2^	BMI ≥ 28 kg/m^2^	*P* value
No. of patients	481	50	314	83	33	
Male/Female (%)	193/288 (40.1/59.9)	15/35 (30/70)	116/198 (36.9/63.1)	40/43 (48.2/51.8)	21/12 (63.6/36.4)^*^	0.004
Age (years)	36.87 ± 11.34	28.28 ± 10.80^*^	36.63 ± 10.54	42.49 ± 11.90^*^	37.88 ± 10.19	3.6E-11
BMI (kg/m^2^)	22.75 ± 3.59	17.34 ± 0.89^*^	21.85 ± 1.74	26.15 ± 0.83^*^	31.03 ± 2.52^*^	3.19E-45
SBP (mmHg)	128.69 ± 18.08	122.96 ± 16.64^*^	128.75 ± 18,50	131.24 ± 18.03	130.03 ± 14.93	0.036
DBP (mmHg)	80.48 ± 12.30	76.12 ± 10.13^*^	80.44 ± 12.61	82.69 ± 12.38	81.91 ± 11.00	0.014
Hypertension (%)	125/280 (26.0/74.0)	6/44 (12/88)^*^	83/230 (26.4/73.6)	28/55 (33.7/66.3)	7/26 (21.2/79.8)	0.043
Gross hematuria (%)	134/459 (29.2/70.8)	23/26 (46.9/53.1)^*^	95/207 (30.3/69.7)	11/78 (14.1/85.9)^*^	4/30 (13.3/86.7)^*^	1.1E-5
eGFR (ml/min per 1.73m^2^)	85.92 ± 32.28	103.96 ± 31.92^*^	84.95 ± 32.60	79.53 ± 28.92	88.98 ± 30.58	6.3E-5
UA (μmol/L)	368.28 ± 118.72	345.67 ± 114.99	360.49 ± 115.41	389.89 ± 115.62^*^	417.39 ± 145.86^*^	0.004
SCr (μmol/L)	94.65 ± 42.36	77.39 ± 31.16^*^	95.57 ± 42.68	101.95 ± 45.19	92.90 ± 41.72	0.005
Proteinuria (g/24 h)	2.06 ± 2.26	1.57 ± 1.75	1.90 ± 1.89	2.83 ± 3.47^*^	2.61 ± 2.40^*^	0.005
Hb (g/L)	131.55 ± 21.19	126.48 ± 19.01	130.03 ± 20.72	136.59 ± 22.28^*^	141.33 ± 21.97^*^	0.002
Alb (g/L)	38.59 ± 7.50	38.32 ± 7.30	38.43 ± 7.41	38.89 ± 8.65	39.72 ± 5.68	0.581

*Abbreviations*: *BMI* body mass index; *SBP* systolic blood pressure; *DBP* diastolic blood pressure; *eGFR* estimated glomerular filtration rate;

*UA* uric acid; *SCr* serum creatinine; *Hb* hemoglobin; *Alb* albumin

^*^, *P* value < 0.05 compared with control group. Patients with normal weight were set as control group

Renal histopathological features of the cohort were described in Table 2. Among a total of 481 patients, 432 had pathology slides available for review. Of which, 390 patients had Oxford Scores report. Interstitial fibrosis was present in 68.8% of IgAN patients. Of these 297 patients with interstitial fibrosis, mild interstitial fibrosis was seen in the vast majority of patients (82.2%). Furthermore, when the association with BMI and pathological characteristics in IgAN patients was assessed, overall no significantly clinical difference among the four BMI groups were seen although *P* value of vasculopathy was less than 0.05.

**Table 2:** Pathological characteristics at renal biopsy

	Total	BMI < 18.5 kg/m^2^	18.5 ≤ BMI<25 kg/m^2^	25 ≤ BMI<28 kg/m^2^	BMI ≥ 28 kg/m^2^	P value
Oxford Score	390	43	260	67	20	
M0/1 (%)	178/212 (45.64/54.36)	15/28 (34.88/65.12)	121/139 (46.5/53.5)	32/35 (47.76/52.24)	10/10 (50/50)	0.5
E0/1 (%)	243/147 (62.31/37.69)	26/17 (60.47/39.54)	158/102 (60.8/39.2)	42/25 (62.69/27.31)	17/3^*^ (85/15)	0.194
S0/1 (%)	221/169 (56.67/43.33)	33/10^*^ (76.74/23.26)	143/117 (55/45)	32/35 (47.76/52.24)	13/7 (65/35)	0.018
T0/1/2 (%)	327/54/9 (83.85/13.85/2.30)	35/6/2 (81.40/13.95/4.65)	214/40/6 (82.3/15.4/2.3)	60/6/1 (89.55/8.96/1.49)	18/2/0 (90/10/0)	0.684
Pathologic lesions	432	44	283	75	30	
Global sclerosis %	11.21 ± 15.31	8.63 ± 12.63	11.72 ± 15.82	10.60 ± 14.43	11.58 ± 16.66	0.628
Segmental sclerosis %	6.82 ± 11.83	4.58 ± 6.86	7.22 ± 13.00	5.77 ± 8.63	8.65 ± 12.90	0.788
Interstitial fibrosis (%)						
None	135 (31.25)	18 (40.91)	92 (32.51)	16 (21.33)	9 (30)	0.134
Mild (< 30)	244 (56.48)	21 (47.73)	153 (54.06)	52 (69.33)	18 (60)	0.065
Moderate (30–60)	47 (10.88)	4 (9.09)	34 (12.01)	6 (8)	3 (10)	0.756
Severe (> 60)	6 (1.39)	1 (2.27)	4 (1.41)	1 (1.33)	0	0.879
Crescents %	8.04 ± 13.677	2.61 ± 2.50	2.32 ± 2.38	7.55 ± 13.62	8.19 ± 12.01	0.704
Vasculopathy (%)	202 (46.76)	12 (27.27)^*^	130 (45.94)	46 (61.33)^*^	14 (46.67)	0.004

^*^, *P* value < 0.05 compared with control group. Patients with normal weight were set as control group

### No relationship between BMI and renal progression and outcomes

In the follow-up cohort, 36 patients out of 295 patients had adverse renal outcomes, of which 3 patients were started on dialysis and 33 patients had 30% reduction in eGFR.

We first investigated the association of BMI with the progression of IgAN. In order to exclude the influence of confounding factors on the progression of IgAN, we set normal BMI group as the reference group and conducted propensity-score matching analysis (Fig. 1). By comparing the eGFR and proteinuria at the time of renal biopsy and last follow up visit in the follow-up matched cohort, there were no significant differences in eGFR and proteinuria between this two time points. Therefore, BMI did not show a significant effect on the progression of IgAN (Table 3). Furthermore, medical treatment to IgAN patients did not show significant effects on renal outcomes in the matched group (Table 3),

**Table 3:** Demographic, clinical, and laboratory characteristics at renal biopsy and treatment

Total		Model 1			Model 2			Model 3		
		< 18.5	18.5–25	*P* value	18.5–25	25–28	*P* value	18.5–25	≥28	*P* value
No. of patients	295	37	37		48	48		12	12	
Follow-up time (months)	45.4 ± 26.6	36.3 ± 24.0	43.3 ± 23.6	0.158	41.5 ± 23.1	43.9 ± 34.3	0.682	40.4 ± 28.2	41.5 ± 21.6	0.810
eGFR (ml/min per 1.73 m2)	88.82 ± 31.57	103.84 ± 34.25	99.83 ± 34.91	0.406	83.99 ± 33.18	83.75 ± 29.88	0.809	76.32 ± 41.11	87.41 ± 30.83	0.722
Proteinuria (g/24 h)	1.60 ± 8.78	0.60 ± 0.46	0.74 ± 0.69	0.567	0.84 ± 0.82	1.01 ± 0.85	0.105	1.22 ± 0.99	1.39 ± 1.43	0.786
Treatment										
P	17	2	2	1	4	2	0.677	0	0	1
P + CTX	1	1	0	1	0	0	1	0	0	1
P + LEF	5	1	1	1	0	1	1	0	0	1
ACEI/ARB	70	10	8	0.787	8	14	0.224	3	2	1
P + ACEI/ARB	81	6	12	0.173	14	11	0.642	4	5	1

*Abbreviations*: *P* prednisone; *CTX* cytoxan; *LEF* leflunomide; *ACEI* angiotensin-converting enzyme inhibitors; *ARB* angiotensin receptor antagonist

Model 1, 2 and 3 were performed by propensity-score matching. The matching covariates were age, sex, eGFR and proteinuria. The control group were patients with normal weight (18.5 ≤ BMI<25 kg/m^2^), and the matching group were patients with low-weight (BMI<18.5 kg/m^2^), overweight (25 ≤ BMI<28 kg/m^2^) and obese (BMI ≥ 28 kg/m^2^)

**Fig. 3:**
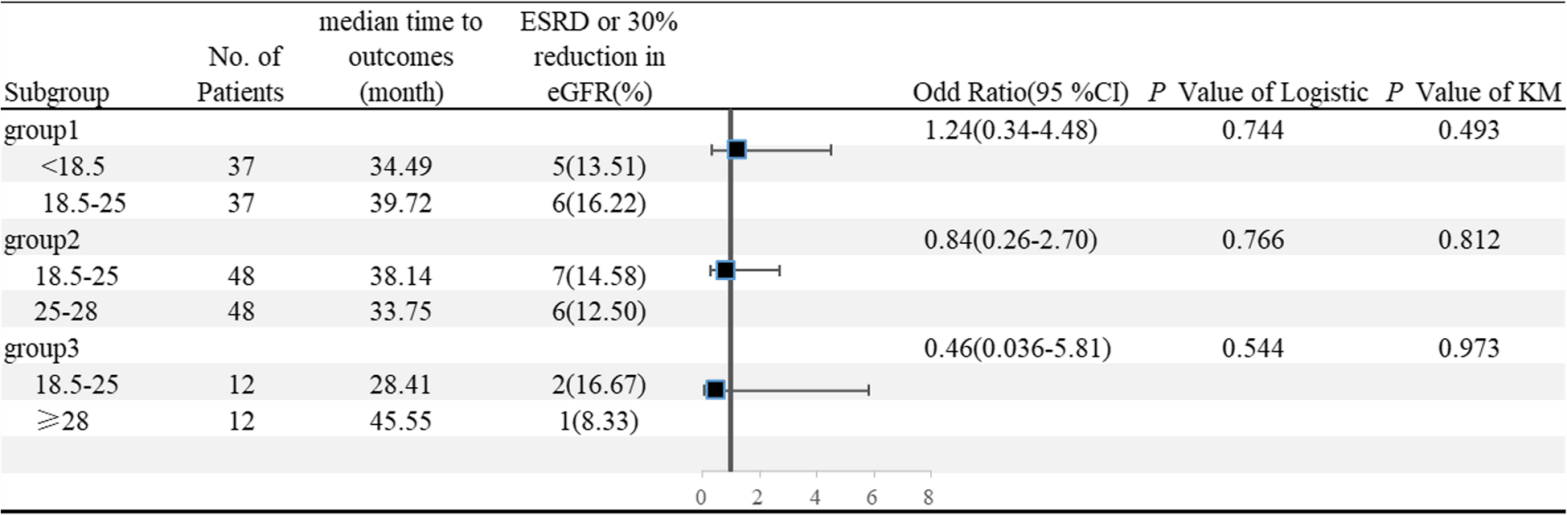
Forest plots of BMI and renal outcomes. Group 1, 2 and 3 all belonged to the follow-up matched cohort. *P* value of Logistic and KM were analyzed by logistic regression and Kaplan-Meier survival analysis

Then we analyzed the predictive value of BMI to outcomes in the ‘follow-up matched cohort’ (Fig. 1). Using Kaplan-Meier survival and logistic regression analysis, we found BMI was not independently predictor for the renal outcomes **(**Fig. 3**)**.

### Interstitial fibrosis was associated with BMI and progression of IgAN

**Fig. 4:**
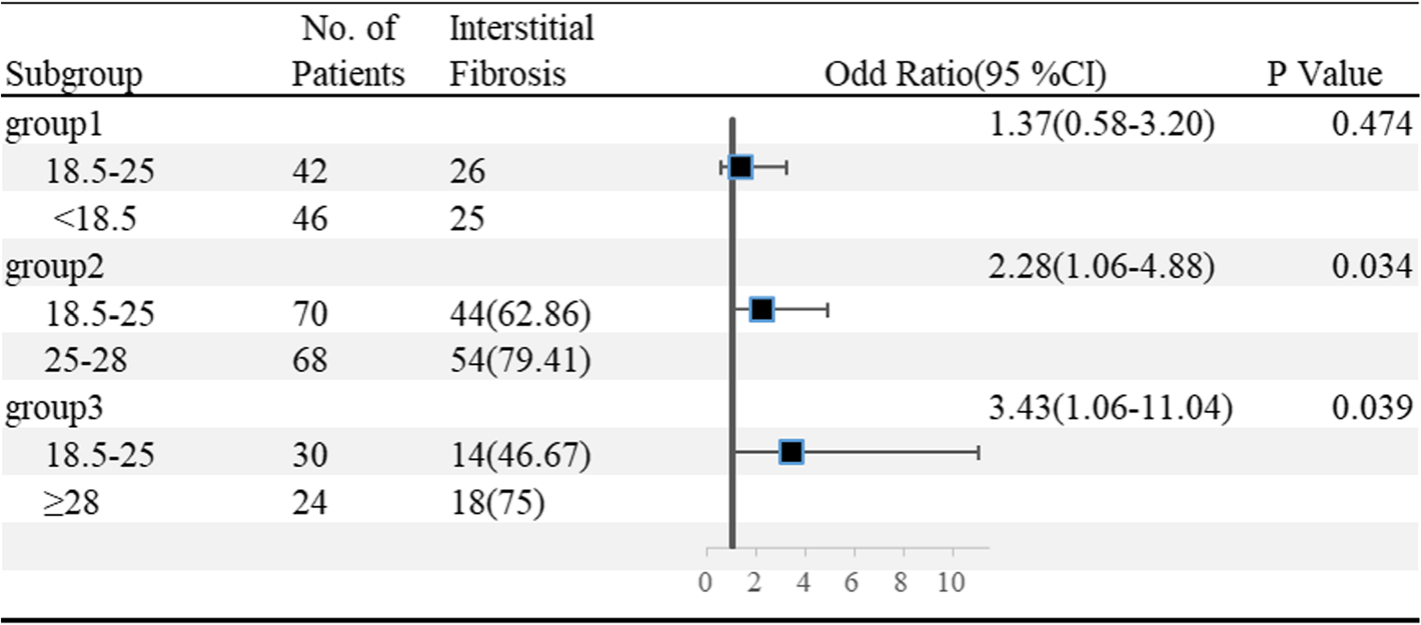
Forest plots of BMI and interstitial fibrosis. Group 1, 2 and 3 all belonged to the based matched cohort. The patients with normal weight (18.5 ≤ BMI<25 kg/m^2^) were set as reference. Finally, 48 underweight patients matched 48 normal weight patients in group 1, 75 overweight patients matched 75 normal weight patients in group 2, and 32 obese patients matched 32 normal weight in group 3. In the Logistic analysis, interstitial fibrosis was defined as the event and the factor was BMI

In the ‘based matched cohort’, we studied the association between BMI and interstitial fibrosis (Fig. 4). 48 underweight patients were matched to 48 normal weight patients, 75 overweight patients were matched to 75 normal weight patients, and 32 obese patients were matched to 32 normal weight patients. Compared with the normal weight patient (the reference group), overweight patients (OR, 2.28; 95%CI: 1.06–4.88; *P* = 0.034) and obese patients (OR, 3.43; 95%CI: 1.06–11.04; *P* = 0.039) were associated with higher risk of interstitial fibrosis. Higher BMI was associated with increased incidence of interstitial fibrosis. However, there was no significant difference in the incidence of interstitial fibrosis between underweight and normal weight patients (OR, 1.37; 95%CI: 0.58–3.20; *P* = 0.474).

**Fig. 5:**
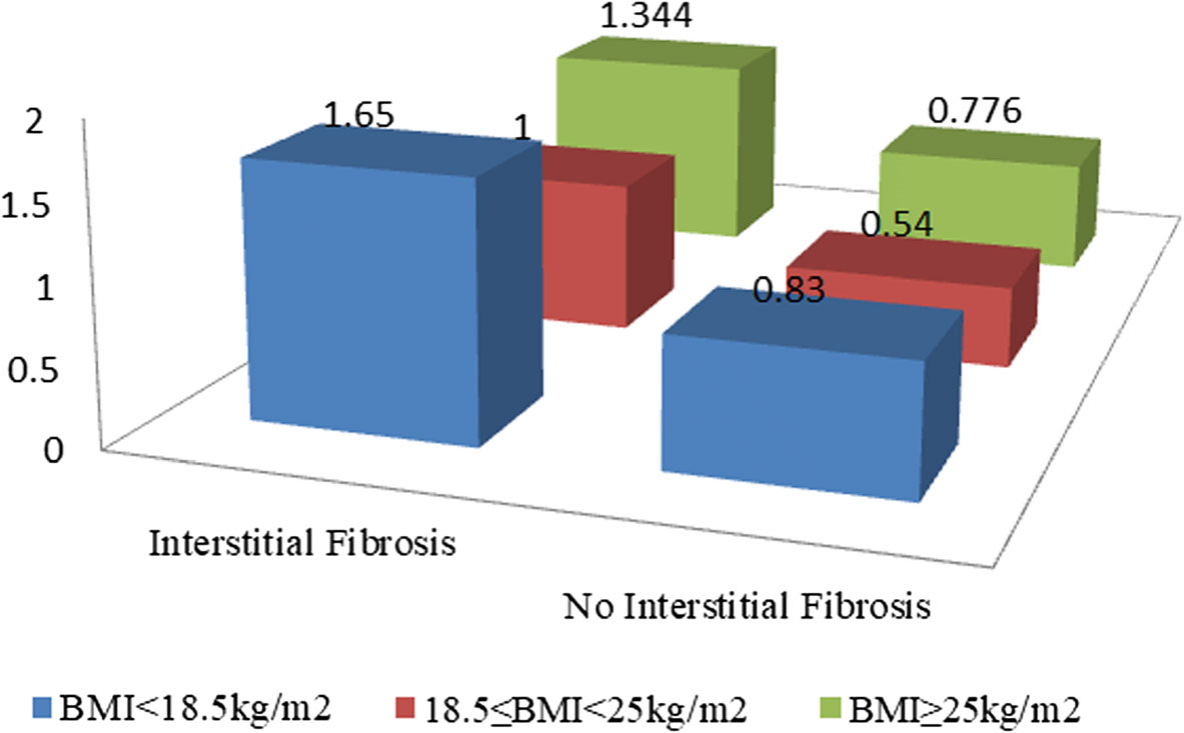
The analysis of additive effect of BMI and interstitial fibrosis on renal outcomes. X-axis standed for interstitial fibrosis, Z-axis standed for subgroups of BMI, and the Y-axis standed for the OR value. Patients with interstitial fibrosis and normal weight were set as the reference. The ORs were 1.65 (95%CI: 0.49–5.61; *P* = 0.42), 1.34 (95%CI: 0.51–3.5; *P* = 0.55), 0.83 (95%CI: 0.10–7.07; *P* = 0.86), 0.54 (95%CI: 0.20–1.46; *P* = 0.23), and 0.78 (95%CI: 0.16–3.71, *P* = 0.75), respectively

Next, we assessed whether interstitial fibrosis and BMI had an impact on renal outcomes in the follow-up cohort. The patients with normal weight and interstitial fibrosis were set as the reference group. For additional interactions of BMI and interstitial fibrosis, as shown in Fig. 5, the ORs of patients with interstitial fibrosis were higher than patients without interstitial fibrosis. In addition, patients with overweight and underweight both had higher ORs than patients with normal weight. This trend demonstrated that interstitial fibrosis and BMI possibly accelerate the progression of IgAN.

## Discussion

Current literature has shown inconsistent results on associations between BMI and progression of IgAN. Some studies indicated that BMI was an independent risk factor of 1.5-fold increase in SCr [10] and progress in IgAN [11]. However, Berthoux et al. [13] did not reveal that BMI was a predictor for IgAN progression even though the patients with elevated BMI was associate with worse presentation at diagnosis and worse clinical outcomes. Elevated BMI appeared to accelerate hypertension, proteinuria and renal lesions, but had no direct effect on IgAN progression.

Our study was undertaken with two purposes: (1) to determine if BMI is an independent risk factor of progression in IgAN; (2) to determine effects of BMI on IgAN and if BMI works on IgAN directly. To accomplish this, we analyzed data of 481 patients from the Southwest China using propensity-score matching.

Our findings did not yet illustrate that BMI worked as the independent risk factor for the progression of IgAN. Consistent with previous researches, elevated BMI was likely associated with worse renal function and clinical outcomes. Mechanisms could explain these findings are as follows. When the metabolic state changes, immune cells which are found in adipose tissue such as macrophages are highly responsive [19]. Hypertrophic adipocytes are more susceptible to inflammatory and inflamed adipocytes are accessible to attract macrophages, which both compose a cycle between adiposity and inflammation [19]. In addition, obesity is associated with hyperleptinaemia, which contributes to a decline in eGFR and pathological processes, particularly fibrosis [19]. We still agreed with Berthoux’s opinion that BMI had no direct effect and can’t predict the progression and clinical outcomes of IgAN directly. It was possible that high BMI indirectly accelerated the progression of IgAN by inducing metabolic syndrome on patients for example [20]. Among all the factors which contributed to the metabolic syndrome, hypertension, high fasting glucose, hypertriglyceridaemia [20] and hyperuricemia [21] were strongly associated with the development of ESRD.

Our study also found that patients with high BMI with more severe interstitial fibrosis were more possibly to progress to the worse outcomes. A cross-sectional study [22] also supported this conclusion, which showed that interstitial fibrosis was the prominent kidney lesions in patients with metabolic syndrome [22]. Metabolic syndrome was also one of the mechanisms of obesity inducing adverse sequelae in cell and organ systems [19]. In addition, hyperuricemia was shown to be associated with interstitial fibrosis at the early stage of IgAN [21]. Higher BMI was also associated with higher plasma uric acid level in this study.

Our study supported and strengthened the important role of BMI in the progression of IgAN. Obesity increased interstitial fibrosis frequency and consequently accelerated the progression and outcomes of IgAN, frequently accompanied by hyperuricemia. Focusing on these factors for patients with elevated BMI might be a potential therapeutic strategy to improve clinical outcomes of IgAN in clinical practice.

Our study, to our knowledge, is the first study assessing the association between high BMI and IgAN in China. It has several strengths. First, propensity-score matching analysis was able to minimize the effect of confounding factors in order to better understand the effect of BMI on IgAN progression. Second, we were able to establish the association between BMI and interstitial fibrosis using a number of statistical analyses such as stratified analyses, propensity score-matched subgroup analyses and survival analyses. Our findings recommended that interstitial fibrosis should be seriously considered in patients with elevated BMI.

The potential limitations should also be taken into account. First, the number of patients with BMI more than 28 kg/m^2^ was relatively small, which may not result in adequate statistical power in this subgroup analysis. Second, a few participants had no pathological slides. Lack of this data may affect the result of OR. Third, this study was a single center, exploratory and retrospective study. Therefore, the results should be validated by further larger studies.

## Conclusions

In conclusion, high BMI and interstitial fibrosis were associated with progression of IgAN. Interstitial fibrosis appears to be common in IgAN patients with elevated BMI.

## Abbreviations

BMI: Body mass index
CI: Confidence interval
CKD: Chronic kidney disease
eGFR: Estimated glomerular filtration rate
ESRD: End stage renal disease
IgAN: IgA nephropathy
OR: Odds ratio
RTS: Renal treatment system
SCr: Serum creatinine
SLE: Systemic lupus erythematosus
UA: Uric acid
